# A pilot mixed-methods evaluation of a dance-based wellbeing programme in a women’s prison

**DOI:** 10.1177/17455057261447141

**Published:** 2026-05-27

**Authors:** Connor Leslie, Wendy Dyer, Kathryn Cassidy, Liz Pavey

**Affiliations:** 1 School of Humanities and Social Sciences, 373113 Northumbria University, Newcastle Upon Tyne, Tyne and Wear, UK; 2 School of Geography and Natural Sciences, 410935 Northumbria University, Newcastle Upon Tyne, Tyne and Wear, UK; 3 School of Design Arts and Creative Industries, Northumbria University, Newcastle Upon Tyne, Tyne and Wear, UK

**Keywords:** mental health, wellbeing, prisons, women, dance, dance-based intervention, mixed-methods research

## Abstract

**Background:**

Engagement in dance has been shown to enhance wellbeing, confidence, and social connection, yet little research has explored its potential in women’s prisons. Women in custody experience disproportionately high levels of mental health and social care needs, highlighting the importance of developing cost-effective, creative interventions.

**Objectives:**

This study examined the impact of a one-week dance-based wellbeing programme delivered by Dance United Yorkshire (DUY) at HMP/YOI Styal on participants’ mental health, confidence, and interpersonal relationships.

**Design:**

A mixed-methods, realist-informed pilot evaluation explored what worked, for whom, in what circumstances, and why.

**Methods:**

Twelve women participated in a five-day dance programme culminating in a public performance. Data were collected through participant observation, semi-structured interviews, focus groups, and validated psychometric tools (WEMWBS and Rosenberg Self-Esteem Scale) administered at four time points (pre, post, 2-day, and 4-week follow-up).

**Results:**

Quantitative findings indicated increases in wellbeing and confidence immediately following the programme, with partial retention at four weeks. Thematic analysis identified three key mechanisms: working through challenging emotions, developing individual agency and confidence, and strengthening positive peer relationships.

**Conclusion:**

This pilot study provides preliminary evidence that short, arts-based dance interventions can enhance mental wellbeing among incarcerated women and merit further multi-site evaluation.

## Introduction

Participating in dance has long, and consistently, been found to shape embodiment, identity, belonging, self-worth, aesthetics, affective responses and creativity.^1^ Staricoff^2^ argued that engaging in the arts alongside health and social care can improve quality of life. The impact on mental wellbeing has also been documented showing the psychological benefits of dance as a potential mental health intervention.^3^ Further research^4^ carried out a review of twenty-three dance programmes and found overwhelmingly positive results, including improvements in self-confidence and self-esteem. Systematic reviews focusing on specific populations demonstrate overwhelmingly positive findings. For example, dance has been found to be beneficial for the physical and psychological wellbeing of children and young people^5^ and older adults.^6^

Less research has focused on investigating the effects of dance in prisons.^7^ The Inspiring Futures publication^8^ describes positive findings from an arts programme including music and drama delivered across various prisons and populations (mainly male prisons). Dance was not however included in the interventions delivered.

While the results of dance-based programmes are positive, evaluation within prison settings is minimal. Page, Chamberlain, and Gratton^9^ evaluated an arts-based programme delivered in His Majesty’s Prison (HMP) Stafford, part of which included dance. It was found that the programme improved physical and mental wellbeing within the male prisoner participants. Nevertheless, there is currently a clear lack of research on dance-only programmes in general and their impacts in women’s prisons, in particular.

There has been significant recent focus on women in the criminal justice system including prison (e.g. ^10–14^). These have shown the unique experiences women face within prison, and their ongoing issues with mental health problems. A review into the health and social care of women’s prisons discusses how:

Women in prison have disproportionately higher levels of health and social care needs than their male counterparts in prison and women in the general population. High numbers of women in prison experience poor physical and mental health and many are living with trauma.^15^

This pilot mixed-methods evaluation was carried out in collaboration with Dance United Yorkshire (DUY) to evaluate their five-day dance programme in HMP/YOI Styal. DUY have delivered a programme of free (at the point of delivery) dance interventions, outreach workshops, and regular group activities across Bradford in West Yorkshire for twelve years, helping to bridge gaps in statutory health and social care provision. HMP/YOI Styal is a prison and young offender institution in Wilmslow, Cheshire, for women aged eighteen and over. According to the most recent His Majesty’s Inspectorate of Prisons (HMIP) unannounced inspection report^16^ the prison has an operational capacity of 422, receives approximately ninety women/month and releases approximately seventy-four/month. With regards to the healthy prison outcome measures (including safety, respect, purposeful activity, and rehabilitation and release planning), while some have reduced slightly from good, all remain ‘reasonably good’. The annual report by the Independent Monitoring Board^17^ describes the prison having,

A complex population of offenders consisting of short sentence (60%), and long-term prisoners (40%), including lifers. It also has a significant number of prisoners who have been recalled, many of whom have issues with substance misuse and mental health.

The evaluation explored the impact of a short dance intervention on women prisoners’ mental health and wellbeing. As an early stage evaluation, the study aimed to understand if, how, and why a dance-based programme impacts on women in prisons’ mental health and well-being. We explored whether and how a short dance programme might be effective by gathering preliminary evidence on impact (mental health and wellbeing outcomes), mechanisms of change (“what works, for who, in what circumstances, how and why”) and acceptability and feasibility (recruitment, participation, missing data, and challenges in prison context).

## Methodology

To understand the impact of this complex intervention and participant group we adopted a broad Scientific Realist^18^ informed framework, which asks ‘what works, for who, in what circumstances, how and why’. A multi-method data collection approach (including longitudinal participant observation, interviews and focus groups, and psychometric tools/structured questionnaires) was used and this data was then triangulated during the analysis phase of the research. The reporting of this study was informed by the RAMESES II standards for realist evaluation, but adapted to suit the complex, exploratory, and arts-based nature of the research.^19^

Ethics approvals were obtained from the His Majesty’s Prison and Probation Service (HMPPS) National Research Ethics Committee (2024-116) and Northumbria University Department of Social Sciences Research Ethics Committee (7194). The inclusion criteria were that they were partaking in the DUY programme at that time.

Twelve women participated in the DUY programme, and all self-selected to be involved after it was advertised using the prison’s internal computer system. The women ranged in age from twenty-one to forty-seven, with a mean age of thirty-six years old. One woman had partaken in the DUY programme before. All twelve women agreed to partake in the research (total population sampling).

At the start of the programme, the Dance Artist introduced the researcher and the study. The researcher explained the aims of the study and what would be involved. The choice not to engage with the research including with the participant observation element was emphasised and that in this circumstance no observations would be recorded, or interviews, questionnaires, or focus groups requested for that individual. The consent form was verbally explained alongside a paper copy, and written consent obtained. The DUY programme lasted 5 days (Monday to Friday), however the research was conducted over one month.

### Participant observation

One member of the research team participated in the programme with the women for the whole week. This included all aspects of the programme, from warm-ups and practices, to the final performance. The final performance was in front of their peers (fellow women in the prison), and staff. Approximately 150 people watched the final performance. Throughout the week, this member of the research team kept an ethnographic fieldwork diary, noting their own and the women’s experiences and some initial analysis and emergent themes and ideas based upon these observations.

While participant observation has several benefits (deep understanding, rich qualitative data, empathy and insight), we also recognise it has potential limitations including researcher bias and the observer effect. To counter bias, the ethnographic fieldwork diary was analysed by the research team including with expertise in the arts, psychology, criminology, and human geography. To minimise the observer effect, we focused on building trust and embeddedness, although we would also point to research which criticises the criticisms of the observer effect.^20^

### Interview and focus groups

Semi-structured interviews with all twelve women were undertaken halfway through day one. The aim of these interviews was to better understand why the women wanted to engage in the programme, and what they were hoping to get out of it.

Two-days after the final dance performance, two focus groups took place, each with four women. The reason for the shift from one to one interviews to focus groups was due to the size of the room provided by the prison to carry out interviews. This change was discussed with both groups of women, and all said they were happy to take part in focus groups. A final focus group with eight of the women took place four-weeks after the final performance.

### Psychometric tools/Structured questionnaires

To assess mental health and wellbeing The Warwick–Edinburgh Mental Well-being Scale (WEMWBS) was used, which has been found to have good reliability.^21^ To assess confidence and self-esteem, the Rosenberg Self-Esteem Scale was used^22^ which has been shown to consistently have high reliability and validity.^23^ The questionnaires were administered at different time points to measure change over time. While we recognise the time frame available to this research was relatively brief, we did want to test if the questionnaires selected would be sensitive to change.

Data was collected at four time periods:

• **Pre**:
  - Twelve women completed the two questionnaires on Day One of their DUY programme. Ideally this would have happened before any aspect of the programme had begun, but due to time and resource restraints, this was not possible.
  - Semi-structured interviews were also undertaken with the twelve women to understand their motivation for taking part in the programme and what they were hoping to gain from it at the half-way point of day one.
• **Post**:
  - After the end of the five-day DUY programme, after their final performance, eight women completed the two questionnaires. Four women were unable to partake in this stage of data collection, two due to illness and two due to court related activities.
• **Two-Day Follow-Up**:
  - Two days after their final day of the DUY programme, eight women completed the two questionnaires.
  - Two focus groups were undertaken with the women, each with four women. These were used to understand what they gained from the week and how it impacted them. Four women were unable to partake in this stage of data collection due to illness.
• **Four-Week Follow-Up:**
  - Four-weeks after their final day of the DUY programme, eight women completed the two questionnaires.
  - One focus group was undertaken with these eight women to understand how they were feeling a month after finishing the programme. Four women were unable to partake in this stage of data collection, three due to being released and one due to being moved to a different location within the prison estate.

### Data analysis and triangulation

The qualitative datasets were analysed using a thematic analysis approach, which was in-line with that of Braun and Clarke’s six-stage process.^24^ Firstly, through the processes of transcription of the interviews and focus groups and through reading the fieldwork notes based on the participant observation, we *familiarised* ourselves with these datasets. Next, we performed an *initial coding* of this data, which aimed to capture emergent themes/properties. These emergent properties were then compared with ideas and concepts *developed* on the basis of the literature review and specific themes were identified. The themes were then developed further and *reviewed* and finally, the themes were structured into a *narrative*, exploring their interconnections, and linking to the wider academic literature. During these final stages, the qualitative datasets were *triangulated* with the quantitative data collected in the questionnaires, to identify findings which aligned, expanded explanations, and any discrepancies or contradictions which required further exploration, enabling further refinement and developing the final narrative structure presented.^25,26^ The themes (as discussed within the Results and Discussion section) are ‘Working through Challenging Emotions’, ‘Exploring Individual Agency & Developing Confidence’, and ‘Developing Positive Interpersonal Relations with Others’.

To analyse the questionnaire data, scores were calculated for the WEMWBS (wellbeing) and the Rosenberg Self-Esteem Scale (confidence), where the higher the score equated to the higher the trait (see Table 1). Unfortunately, no robust statistical analysis could be done on the quantitative data due to the low number of participants and incomplete data points, therefore the quantitative results should be interpreted as preliminary.

**Table 1.** Means and standard deviations for wellbeing and confidence at the four time periods.

​	WEMWBS/Wellbeing mean (SD)	Rosenberg scale/Confidence mean (SD)
Pre	45.17 (13.33)	15.92 (4.12)
Post	60.75 (7.10)	19.25 (3.65)
Two-day follow up	59.00 (8.02)	20.62 (4.17)
Four-week follow up	50.63 (10.77)	17.75 (4.65)

## Results

Below we discuss the findings of the study and the key aspects of what the women experienced across the week.

## Enhancing women’s mental wellbeing through dance

**Figure 1.**
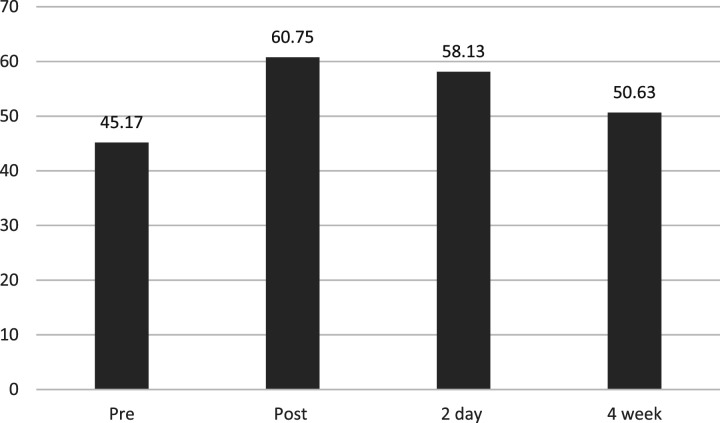
WEMWBS mean scores for wellbeing across the four-time frames.

Our research suggests that DUY’s programme enhanced the participants’ wellbeing. When viewing all participants’ data (see Table 1 and Figure 1), we see an improvement in wellbeing from pre dance, to post dance (mean – 45.17 and mean – 60.75 respectively). While scores for wellbeing do decrease from the peak scores at the post time scale, wellbeing scores at the 2-day follow-up (mean – 58.13), and 4-week follow-up (50.63) are on average higher than where they were at the beginning of the research.

Below we present our findings regarding *how* the DUY programme was able to enhance women’s wellbeing. First, we focus on the ways in which their experiences with DUY’s programme enabled the women to more effectively do emotion work, specifically to work through negative emotions and understand non-linear processes in relation to their emotions as discussed by Hochschild.^27^ Second, we analyse the ways in which the women developed a greater sense of their own agency, which enabled them to build confidence and self-esteem. Third, we explore what the intervention meant for the women collectively, how it enabled them to develop their interpersonal skills and relationships with other women during the programme and the ways in which this generated possibilities for improved relationships between women in prison more broadly.

### Working through challenging emotions

In Hochschild’s^27^ original thesis, a distinction is made between the self-management of ‘emotion work’ and the more public display of emotional labour, often within organisations and workplace settings. It is this self-management of emotions or emotion work within prison settings with which we are concerned here. When observing the women across the week, it was clear that they all went through a non-linear emotional journey.

On the first day of the programme, the women were visibly nervous when they walked into the room where the programme was being delivered. In the interviews, some of the women explained that this anxiety was generated primarily by concerns over meeting new people, ‘[I was worried about] meeting new people, not the dancing, meeting people’ (participant ten). Whereas others were also worried about what the programme itself might entail, ‘I was a bit anxious [this morning] to see who would be here and what we’d be doing’ (participant five).

Some of this anxiety was visibly reduced on the first day, with a clear shift in how the women were feeling after lunch. Some women had changed clothes into sportswear, and you could see a physical difference in them, they were standing taller, they were smiling and laughing, a clear difference from how nervous they initially seemed. The member of the team participating in the programme noted that there was a positive energy in the air and the women were smiling and laughing and generally excited for the week. This was also noted by participants in the follow-up interviews.

My mood switched; I just felt the energy straight away it was so positive it was so welcoming. I didn’t bring my problems through that door. Then I’d come home [to their prison wing] and look what I’d done and what I’d achieved in that one session stuff like that. It was an energy change (participant eleven).

The participants described being able to take the energy and what they had achieved away with them and that this led to a change in them as individuals as well. However, this level of positive energy and enthusiasm was not sustained and by the third day of the programme the women started to feel physically and emotionally tired and they had also started to worry about the final performance. This precipitated arguments amongst the women. This was due to both physical exhaustion (most of the women did not engage with daily physical activity before the programme), and the worry that the final performance was nearing. However, later the same day, the women learnt the final part of the dance in order to practice the final performance that afternoon. While the women were still exhausted, they were able to come together and focus on learning the final parts of the dance. After the practice performance went well, we were able to observe that the women looked more confident, standing taller with smiles. This is seen in Tuckerman’s model of group development,^28^ where the women went through the process of Forming, Storming, Norming, Performing, and Adjourning.

The women showed that they were able to maintain this focus even when circumstances meant that four of the participants were away on the day of the final performance and the DUY artists had to step in. The final performance ended to huge applause from the audience and many women and members of the audience were crying. There was an overwhelming sense of positivity within the group even after only a few hours.

Every woman discussed how they already felt a positive impact even at the early stages and were glad have participated in the programme. Participant ten described being worried as she was not confident, but recognised that she was already becoming more confident, ‘Yeah because I’m not that confident, it worries me now, but it’s already bringing me out of my shell more’. Participant three felt that her improved outlook was visible outside of the sessions, ‘I look happier around the house [their prison wing] already’.

Not only were the women seeing improvements in themselves, but they frequently talked about seeing improvements in the women around them, most of whom they had only met that week.

I see the women smiling. When we went to the first meeting last Monday this girl wasn’t even speaking, and now I see her she’s got her top off like Billy Elliot, you know what I mean, she’s loving life now (participant ten).

Participant eleven suggested that these improvements related to their anxieties about the choice to participate in the programme being alleviated as she ‘could see in other people, not just myself, people began to feel that they made the right choice and left here better than they came’. It was clear that they also viewed the experience positively as a result of overcoming these barriers to participation, ‘I’m so proud of us because we all overcame something. All of us’ (participant four) with participant five describing the whole experience as ‘very rewarding’.

The women also discussed the benefits of the programme on their mental health. Throughout the week, the women had openly discussed the mental health struggles they have experienced (both within and outside of the criminal justice system) and this made them conscious of the improvements that the programme precipitated. ‘That’s the first week I’ve had that I’ve not felt depressed in a long time’ (participant three). Another noted the combination of the positive impacts on her mental and physical health and the potentialities for the programme to have the same impact on many other women in the institution. ‘How good it is for your mental health and your body, you’re pushing your body, it’s really really good for you and it should be done more often. So many girls would benefit’ (participant eight).

In some ways the women recognised that the programme eased the ‘tightness’^29^ of the carceral regime and enabled them to forget their surroundings, which had a positive impact on their mental health and wellbeing. ‘Even though we are in jail, last week I felt freedom […] I felt free’ (participant eleven). ‘For them few hours it felt like we were outside’ (participant eight). Our research showed that the relationships with and attitudes of the DUY instructors were crucial in alleviating the carcerality of the setting. ‘The way we were taught, they treat us like we were a dance school. I would happily go around the prison and explain to the girls what we did and how it made us feel’ (participant eight). We see that being treated like they were in a different setting enabled them to feel as if they were with participant 8 being so confident in how this had made her feel that she was willing to go and express this to other women. It could also be that the positive emotional state of the dance artists transferred to the women in emotional transference to boost their wellbeing.

One of the key skills for emotion work that the women described developing was patience, which they understood to be important in their specific prison context. Participant four stated she had ‘learned patience’, with participant six reflecting that ‘In here everything is a waiting game, all you do is wait, you need patience’, and participant four describing patience as ‘a virtue in here’. This patience came from learning the dance, which at times could be challenging, but they learnt patience to be able to do it, something they continue to practice at the four-week follow up, with the women expressing it had help them remain calm within the prison setting. This learned patience could have a long-term positive effect on the women. While no research has specifically been done within a UK prison population, there has been found to be a link between increased patience and increased mental health.^30^

Their understanding of the impact that the programme had on their mental health and wellbeing also shaped their thinking about participating in similar activities in the future to sustain and even enhance these outcomes. For example, after only one morning participant five explained that she had ‘already been thinking about dance classes when I get out’ and participant one said she would, ‘sign up to something like this again because I quite enjoy it’. In this section we have described the impact of the programme on the women’s ability to do emotion work; specifically, to work through negative emotions like anxiety, anger and frustration, focus on complex and difficult tasks and develop patience, in order to enhance their emotional and mental wellbeing within the carceral environment of the prison. Next, we explore how the programme enabled them to further develop their confidence.

### Exploring individual agency & developing confidence

Many of the women described improving their confidence as a key outcome they were hoping to achieve as part of participating in the programme. Some of them simply recognised that they were lacking in confidence, for example participant three stated that ‘[I decided to do this] cos I’ve got no confidence really’*.* However, for others this lack of confidence represented a loss – something that they had earlier had and wanted to get back. ‘The other reason I wanted to do it is to build my confidence again. If you haven’t noticed I’m quite quiet with everyone. I’m not usually as quiet’ (participant two). Another participant noted being more aware of the positive impact as she had been involved in dance before. ‘I’ve done dance on the outside, so I thought I’d give it a go, boost my confidence levels’ (participant twelve).

**Figure 2.**
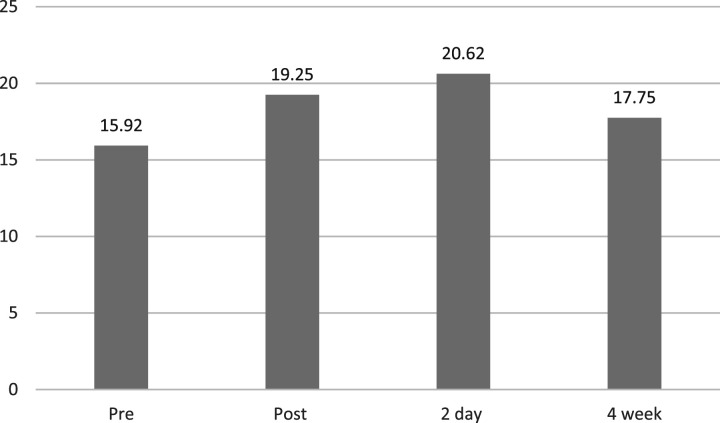
Rosenberg scale mean scores for confidence across the four-time frames.

When viewing all participants’ data (see Table 1 and Figure 2), we see an improvement in wellbeing from pre dance, to post dance (mean – 15.92 and mean – 19.25 respectively). Interestingly, the women’s confidence scores were higher at the 2-day follow up than at post (mean – 20.62). When speaking to the women at a later date, they believed that this may be due to fellow women who had attended the final performance coming up to them at the weekend and complimenting them on the dance. At the 4-week follow up (mean – 17.75), the score did reduce from the two-day follow up however it was still higher than the pre scores. One explanation for this is could simply be a reduction due to the women going back into their standard regime in prison.

When comparing the confidence post scores to the pre scores, two participants scores decreased, one stayed the same, and five increased. One explanation for the two scores that decreased is one of the women had partaken in the DUY programme before and had therefore felt more confident going into it. The other women whose score decreased started with a very high confidence score, and while the score did go down, she still ended with very high confidence. Furthermore, both of these women had the highest pre confidence scores.

When comparing the confidence two-day follow up scores to the pre scores, one stayed the same, and seven increased.

When comparing the confidence four-week follow up scores to the pre scores, three participants scores decreased, two stayed the same, and three increased. Interestingly, of the three women whose confidence scores decreased, two did not partake in the final dance, and one started with the highest pre confidence score and had already partaken in the DUY programme.

In the follow-up focus groups, we explored why the women felt more confident in more detail. One of the explanations related to an individual and collective sense of achievement. So much time in the prison was spent doing and achieving little and the week-long programme allowed the women to break with this routine and actually explore what they could do, giving them a greater sense of autonomy and their own agency. Participant six explained, ‘I want to be part of something, it’s nice to be part of something, something to do, as in rather than Groundhog Day, actually achieving something. We are sat there bored out of our brains’. Their understanding of their own agency was enhanced to such an extent that they felt able to use this to write a letter to the governor to demand the programme be provided again due to their positive experience of it.

The feeling of achievement appeared to be enhanced with the external recognition that taking part and the final performance itself gave them. A number of participants articulated that this resulted in others feeling proud of them and also them feeling proud of themselves. Participant ten stated, ‘I’ve never done anything like this before. Make my mum proud.’ Participant eight described the impact the performance had on others, ‘The girls [watching] said it was brilliant. They were really proud’. This sense of achievement, therefore, spread to others in the prison, but also family outside and even external employees. ‘I was [talking] to my other half and he was like fucking hell babe I’m so proud of you’ (participant four). ‘Even my maths teacher couldn’t stop himself from crying’ (participant six).

Overall, the comments from the women in the two-day follow up focus groups confirmed that they were feeling better in themselves. In the final section of this research, we explore this collective experience and particularly the role that the programme played in enabling the women to develop more positive interpersonal relations with others.

### Developing positive interpersonal relations with others

The interviews on the first day of the programme revealed that the women also saw building relationships with others as part of improving their confidence. Participant four stated, ‘I want confidence, it’s a confidence builder, make some new friends, something to be proud of. I’ll be proud of myself.’ There was a sense that this would be an achievement in itself but also that working together would enable them to achieve something (the performance) too. Participant ten explained, ‘The social yeah, do something, achieve something.’

In part, building other relations was part of the desire to relieve the tightness and monotony of prison life described above. Participant six described being motivated, ‘To get to know other people, bit of head change, it’s like ground hog day in here, it’s different.’ However, it was clear in the post-programme focus groups that the programme had done more than just enabling the women to get to know different women in the prison. ‘To be able to take a group of women like us and change how we are and build friendships’ (participant four). There was an understanding that the programme had precipitated a more fundamental change in the women and how they related to one another.

We observed during the programme that the women understood that for the final performance to be successful they would have to support each other in navigating some of the difficult and more complex elements of the routine. Therefore, when participants did complex parts of the dance, the other women would cheer and clap. In addition, when a woman was feeling discouraged, others would stop what they were doing and offer encouragement and support. The women were adept at reading the emotional state of others and providing this support in key moments. However, they also noticed and remarked on when things were going well. At one point during a warmup, one of the women cried from joy, saying, ‘You wouldn’t think we were in jail, look how happy we are.’ This led to the participants all engaging in a group hug; a physical manifestation of the understanding that had developed between them and also something which was spontaneous and unusual in the prison context where physical contact between them is often low/minimal. We saw another physical manifestation of the close and supportive relationships the women had developed before the final performance. The participants huddled together, cheered each other on, hugged one another and told each other how proud they are of them. They were able to achieve this in spite of many visibly feeling nervous; demonstrating they were able to overcome their own anxiety and think of others, reminding them that they were part of a team and not on their own.

One of the things the programme did to improve relations was develop trust between the participants. There was a lift in the dance performance, where all the women had to work together to lift someone above their heads, and carry her for a section of the dance. From observing the women, this lift was never an issue, and when asking the women if they were nervous about lifting/being lifted, the answer was no, as they trusted each other. In reflecting on the programme in the focus groups, the women discussed how teamwork brought them together and had a positive impact on them. With participant six explaining, ‘[it worked because we] lean on each other, be there for each other.’

We were able to observe that in the week, the women were able to develop important relationships. After the performance, before running off to their friends in the audience, the women hugged and went around praising one another. In the first follow-up focus groups, we saw that these relationships remained in-tact and that they enjoyed catching up with each other. Some had experienced issues over the weekend since the final performance, and when they came together they helped solve problems and offered support. We observed a similar pattern at the four-week follow-up, where they demonstrated an eagerness to spend time discussing what had happened in their lives since they last saw each other. The women discussed how they would wave when they saw one another, and many of the participants commented on the importance of these relationships to them. Participant eight described how the programme was able to bridge the many differences between them, *‘We’ve always got that bond between us now. We are all different ages, different background and crimes, we have that bond and can’t take that bond away.’* Participant 9 recognised how these relationships made her feel comforted even though they were formed rapidly, ‘The bonds we have are amazing, for comfort. To start that group on Monday and come to that on the Friday.’

It was also clear from the post-programme data collection that the programme and their participation in it was also a catalyst to developing improved and new relationships with others in the prison. Participant four explained that discussing the performance got conversations with others off to a positive start, ‘People I don’t know are coming up to me talking about it. How good it is.’ The participants gained visibility in the prison in a positive way, ‘All the other girls saying oh I saw you dancing the other day and it was brilliant and loads of people said to them’ (participant five). The reverberations within the prison and the participants’ relationships were still being felt four-weeks’ later with participant six noting, ‘People are still talking about it.’ The programme, therefore, had a positive impact not only on how the women related to each other during the week, but also longer-term and with others within the prison and staff outside of the programme itself. These findings are similar to what has previously been found,^9^ which further supports the importance of smaller programmes within prisons to improve not only the well-being of those who partake in them, but those around them.

## Discussion

In regard to the findings in relation to a broad Scientific Realist^18^ and understanding what worked, the women discussed how the whole programme worked for them. While none of the women were the same, and their journeys were also not the same (with some women coming in with high confidence, some women lacking confidence throughout the week), it is clear from talking to the women that they have all taken something positive away from the experience.

For future research, the key findings from the current study could be explored in more detail. For example, the social bonds the women formed were very apparent. It would be beneficial to explore if these bonds continue beyond the four-weeks to better understand how and why the programme created this impact. While the women all discussed the benefits of the bonds they formed, understanding each personal experience in the long term could tell us more about the long-term benefits and the personal context of them. Furthermore, the women taking an interest in their physical health was apparent at the four-week mark, something found in previous work.^9^ This could be explored in more detail by discussing this at the pre and 2-day follow up and again seeing if this is still apparent beyond the four-weeks. Furthermore, data saturation was not achieved due to the uniqueness of the women and their environment, therefore a longer study could be undertaken to see their changing thoughts over a longer period.

## Limitations

While there were strengths to the study, there were also a few limitations to improve upon with future research and evaluation. A lack of control group (such as women not in prison) would have given more insight into if this programme has more of an impact on those in prison, compared to those out of prison. The key limitation of the current study are the low participant numbers. Furthermore, due to a large percentage of the women not being able to partake in each stage of data collection, for the quantitative data there were only four full data sets. This study highlights some of the complexities of conducting research within prisons. The reason for the low number of full data sets is due to the women missing parts of the programme, mainly due to illness and court related activities. This resulted in many women missing parts of the programme, which could further be investigated into the potential impact of this and how/if it could be avoided. While there were missing data points, it was still important to look at all the data, as it still shows the impact the programme had upon the women. Finally, as data saturation was not achieved, this is also a limitation of the study.

## Conclusion

To conclude, the study aimed to understand the effects of a week-long contemporary dance programme delivered by DUY in HMP/YOI Styal. Wellbeing and self-confidence questionnaires were undertaken by the women at four different time points, with interviews and focus groups provided at three of those times, and participant observation carried out over the entire 5-day programme including final performance. The findings from the current study suggest that the DUY programme delivered to the twelve women in HMP Styal/YOI did have a positive impact on their mental health and wellbeing, specifically their emotional wellbeing. The data suggests that the programme should be provided again. This positive impact could be an additional provision aimed at helping mental health and wellbeing within women’s estates. We have argued that the programme supported improved mental health and wellbeing in three ways: it enabled the women to deal with negative emotions and continue with the activities towards a positive outcome; it built their confidence and sense of agency in a context where they don’t have many other opportunities for this; it enabled them to build positive and supportive relationships with others within the prison based on trust. Overall, the study adds not only key knowledge in understanding methodological approaches to test the impact dance-based programmes have, but also unique contributions to the area.

## Data Availability

An edited data set to protect anonymity is available from the corresponding author upon reasonable request.*
